# Student perception about working in rural United States/Canada after graduation: a study in an offshore Caribbean medical school

**DOI:** 10.12688/f1000research.5927.2

**Published:** 2015-04-23

**Authors:** P Ravi Shankar, Arun K Dubey, Atanu Nandy, Burton L Herz, Brian W Little

**Affiliations:** 1 Xavier University School of Medicine, Oranjestad, Aruba

**Keywords:** Canada, Caribbean, doctors, healthcare, physicians, rural, shortage

## Abstract

**Introduction:** Rural residents of the United States (US) and Canada face problems in accessing healthcare. International medical graduates (IMGs) play an important role in delivering rural healthcare. IMGs from Caribbean medical schools have the highest proportion of physicians in primary care.  Xavier University School of Medicines admits students from the US, Canada and other countries to the undergraduate medical (MD) course and also offers a premedical program. The present study was conducted to obtain student perception about working in rural US/Canada after graduation.

**Methods:** The study was conducted among premedical and preclinical undergraduate medical (MD) students during October 2014. The questionnaire used was modified from a previous study. Semester of study, gender, nationality, place of residence and occupation of parents were noted. Information about whether students plan to work in rural US/Canada after graduation, possible reasons why doctors are reluctant to work in rural areas, how the government can encourage rural practice, possible problems respondents anticipate while working in rural areas were among the topics studied.

**Results:** Ninety nine of the 108 students (91.7%) participated. Forty respondents were in favor of working in rural US/Canada after graduation. Respondents mentioned good housing, regular electricity, water supply, telecommunication facilities, and schools for education of children as important conditions to be fulfilled. The government should provide higher salaries to rural doctors, help with loan repayment, and provide opportunities for professional growth.  Potential problems mentioned were difficulty in being accepted by the rural community, problems in convincing patients to follow medical advice, lack of exposure to rural life among the respondents, and cultural issues.

**Conclusions:** About 40% of respondents would consider working in rural US/Canada. Conditions required to be fulfilled have been mentioned above. Graduates from Caribbean medical schools have a role in addressing rural physician shortage. Similar studies in other offshore Caribbean medical schools are required as Caribbean IMGs make an important contribution to the rural US and Canadian health workforce.

## Introduction

A recent article mentions that rural Americans have limited access to health care ^1^. The authors mention two reasons for this; the first being many Americans, especially in rural areas, are without health insurance, and the second being only 11.4% of the country’s doctors practiced in rural areas in 2005, serving about 20% of the country’s population ( http://depts.washington.edu/uwrhrc/uploads/RHRC_FR125_Rosenblatt.pdf). Family physicians distribute themselves in proportion to the population in both urban and rural areas but doctors of other specialties are much more likely to settle in urban areas.

A study published in 2009 has shown that international medical graduates (IMGs) filled the gaps in requirement of primary care doctors in many rural areas but there were wide differences between various states in America ^2^. The authors concluded that policies to address the mal-distribution of physicians in the United States (US) should also consider the role of IMGs. Critical access hospitals are a federal Medicare (the system of health insurance in the US) category for isolated rural facilities with 15 or fewer beds and IMG physicians play an important and growing role in staffing these hospitals ^3^. Canadian trained physicians migrate both nationally and internationally and IMGs play an important role in providing healthcare in physician losing locations in Canada ^4^. The author concludes that IMGs will be needed in underserved locations for years to come.

A study published in 2013 showed Caribbean IMGs had the highest proportion of physicians practicing in primary care specialties compared to all other categories of graduates ^5^. They make an important contribution to the US primary care workforce. A study in Canada found that around 20% of Canadian IMGs (Canadian citizens or residents who did their medical training overseas) obtained their medical degree from the Caribbean ^6^. A study which examined US citizens who graduated from medical schools outside the US and Canada found that the Central American and Caribbean region graduated more physicians than any other region ^7^. Thus Caribbean medical schools may play an important role in producing primary care physicians for the US and Canada.

Xavier University School of Medicine (XUSOM) is an offshore Caribbean medical school located in Aruba, kingdom of the Netherlands, admitting students from the US, Canada and other countries to the undergraduate medical (MD) course. Students complete the basic sciences in Aruba and do their clinical rotations in the US. The number of students in XUSOM at present ranges from eighty to a hundred students a semester with around twenty new students being admitted each semester, which is less than the number enrolled in the bigger Caribbean medical schools. In addition, the major shareholders of the school are a group of Indian Americans and a large proportion of the student body is of South Asian and Indian origin. There are also students originating from other countries in Asia. Most students in XUSOM are from middle and upper middle class socioeconomic backgrounds. We do not have information on the financial and social background of students in other offshore Caribbean medical schools but believe most of them are likely to be from a similar background. The school also offers a premedical program for high school students to prepare them for admission to the MD program. The premedical program runs for four semesters, while the preclinical MD program is of six semesters. Each semester is of 15 weeks duration.

The studies mentioned in this article from the US and Canada have defined a Caribbean IMG as either a citizen from the US/Canada who had studied in an offshore Caribbean medical school or students from other countries who studied at these schools and eventually plan to practice in the US/Canada. For the purpose of this study ‘IMGs from Caribbean medical schools’ may be considered as undergraduate medical and premedical students from XUSOM who intend to practice in the US/Canada after graduation. We make the assumption that all our students intend to practice in the US/Canada after graduation which may be an overestimation as not all students may do so.

Student perception about working in rural US/Canada has not been previously studied in the institution. Hence the present study was carried out. The study was conducted among premedical and basic science (preclinical) undergraduate medical students to obtain basic demographic information, understand their perceptions regarding why doctors are reluctant to work in rural US/Canada after graduation and conditions to be fulfilled before they would consider working in a rural area, identify possible problems of working in rural areas and obtain possible solutions.

## Methods

The study was conducted among Premedical 1 to 4 semester and undergraduate medical students (semesters 1 to 5) at the Xavier University School of Medicine (XUSOM), Aruba during the month of October 2014. Students during the clinical years of study in the US and Canada were not included. Students were informed about the aims and objectives of the study and invited to participate. They were informed that participation in the study was voluntary and they were free not to participate. Written informed consent was obtained from each participant. The study was approved by the Institutional Review Board (IRB) of XUSOM vide notification XUSOM/IRB/2014/05.

The questionnaire used was modified from that used in a previous study on student perceptions about working in rural Nepal after graduation ^8^. The questionnaire was modified to the US/Canadian context by the authors. Inputs were also obtained from selected faculty members of the institution. The questionnaire used in the study is shown as Supplementary File 1. Information about the semester of study, gender, nationality, place of family residence and occupation of parents were noted. Students’ responses to different questions were noted. Common responses were tabulated. Among the topics studied was whether the strident had lived in a rural area before joining XUSOM, whether they plan to work in rural US/Canada after graduation, and if yes whether they plan to do so immediately after graduation/residency or later in their career. They were also requested to state three important reasons why doctors are reluctant to work in rural areas, and also their opinions regarding how governments can encourage doctors to work in rural US and Canada. Students were also asked whether they felt their medical curriculum prepared them adequately for rural practice and if they felt this was not the case, what modifications they felt were necessary in the medical curriculum. The data was analyzed using descriptive statistics. The percentage and number of respondents mentioning a particular response to a specific question was noted.

Their opinion regarding whether students were aware of the problems of life in rural areas, problems which they anticipate in dealing with the local population, their knowledge of initiatives to address the shortage of doctors in rural US/Canada and in other countries and whether poor return of investment was a factor hindering doctors from working in rural areas was noted. The data was analyzed manually by the authors. The demographic information was analyzed using Statistical Package for Social Sciences (SPSS) version 20 for windows.

## Results

Ninety-nine of the 108 enrolled students (91.7%) participated in the study. Table 1 shows the demographic characteristics of the respondents. Twenty-eight of the 34 premedical students (82.3%) and 71 of the 74 MD students (96%) participated. The number of male and female respondents was approximately equal and 28.3% of respondents were from rural areas. The majority of the student’s parents worked in non health related professions.

**Table 1.** Demographic characteristics of respondents.

Characteristic	Frequency (percentage)
Semester of study Premed 1 Premed 2 Premed 3 Premed 4 MD 1 MD 2 MD 3 MD 4 MD 5	8 (8.1) 4 (4.0) 9 (9.1) 7 (7.1) 24 (24.2) 6 (6.1) 12 (12.1) 15 (15.2) 14 (14.1)
Gender Male Female	48 (48.5) 49 (49.5)
Place of family residence Urban Rural	68 (68.7) 28 (28.3)
Nationality American Canadian Other	37 (37.4) 21 (21.2) 35 (35.4)
Occupation of father Health related Other	27 (27.3) 67 (67.7)
Occupation of mother Health related Other	27 (27.3) 68 (68.7)

Premed – student of the premedical program, MD- student of the medical program

Forty respondents (40.8%) were in favor of working in rural US/Canada after graduation. Ten respondents (mostly from the premedical course) were not sure about whether they planned to work in rural US/Canada at this initial stage. With regard to the duration for which they planned to work in a rural area, 12 stated for most of their career or indefinitely while nine respondents stated for 5 to 10 years while four respondents stated between 1 to 5 years. Eighteen respondents (18.2%) preferred to work in a semi urban or semirural area while eight preferred a rural area. However neither the authors nor the respondents defined what exactly was meant by a rural or a semirural area. The respondents in our study were diverse but no important difference was noted with regard to perspective about rural practice among different subgroups. The number of students was low and this may have been partly responsible. Also the study was not specifically designed to examine for differences in perception about working in rural areas among different subgroups of respondents.

Table 2 shows important conditions which may need to be fulfilled before respondents would consider working in a rural area. Respondents were concerned about housing, regular supply of electricity, internet and telecommunication facilities, and good quality water supply. Family-related factors were mentioned as important and were related to whether the doctor would be able to bring their family to the location, the place should be not very far from his/her hometown, and there should be facilities for education of children. When respondents were referring to proper working conditions they were mainly talking about adequate diagnostic and treatment facilities in the hospital or clinic where they would be working. Respondents also mentioned availability of treatment facilities for themselves and their family if they fall ill is important, and that the practice area should not be extremely cold, the community should welcome individuals from different backgrounds, absence of racism, provide opportunities for personal growth and help with repayment of student loans as necessary conditions. A respondent [Participant 83(P 83)] mentioned, “ *I have lived in a rural area for almost my whole life. I have seen how we have to travel to cities for basic medical services and to help make a change in this situation I would like to work in a rural area*”. Forty-four respondents mentioned that they plan to work in a rural area immediately after graduation while 23 respondents mentioned that they would prefer to do so later in their careers.

**Table 2.** Conditions necessary to be satisfied by the respondents before they would consider working in a rural area.

Condition	Number (percentage)
Good living conditions	20 (20.2)
Safety of the rural environment	17 (17.2)
Higher salary compared to doctors working in urban areas	17 (17.2)
Family-related factors	15 (15.1)
Should be close to a big city	8 (8.1)
Proper working conditions	7 (7.1)

Table 3 shows important reasons why doctors are reluctant to serve in rural areas according to the respondents. Among other reasons mentioned were issues of safety, isolation, slow pace of rural life, racism and greater potential for career and personal growth in an urban area. A participant (P 33) stated, “ *Rural practice is mostly a GP job and most future doctors want to be specialists (including myself). Away from home (if you are from the city). Looks ‘boring’*”. With regard to how the government can encourage doctors to work in rural US/Canada important initiatives/measures mentioned were providing greater benefits and salary to rural doctors as compared to doctors practicing in urban areas (57 respondents), loan forgiveness or help with repayment of student loans (12 respondents), improving the facilities at rural and community hospitals (10 respondents), provide opportunities for professional growth and development for rural doctors (8 respondents), and emphasize ‘educating doctors for rural areas’ (6 respondents). Four respondents mentioned making certain duration of rural service mandatory as has been done in many developing nations. A respondent [participant 98 (P 98)] stated, “ *Make it mandatory for graduates to serve in rural areas and have them serve at least a year or two*”. Seventy-one respondents felt their medical training adequately prepared them for a career as a rural physician while 19 respondents were of the opinion that it did not do so.

**Table 3.** Important reasons why doctors are reluctant to serve in rural areas.

Reason	Number of respondents
Not enough compensation differential compared to doctors working in urban areas	27
Fewer patients and a lower diversity of patients due to lower population in rural areas	14
Lower standard of living	14
Family-related issues	12
Doctors predominantly hail from urban areas	12
Decreased social ties and activities	11
Lack of diagnostic and therapeutic resources	10
Doctors are unfamiliar with the rural lifestyle	10

Many respondents did not answer the question regarding suggestions for re-focusing medical education for preparing doctors for rural practice. Only fourteen respondents (14.3%) answered this question. Among the suggestions mentioned were educating students on how to treat and manage medical conditions in a resource constrained setting, having preceptors with rural experience, initiating clinical exposure for students in rural areas and conducting sessions on cultural diversity and cultural issues in rural areas. When asked whether medical students in the institution were aware of the problems of life in rural areas 43 students (45.4%) were of the opinion that they were aware while 39 respondents stated that students were not aware. Regarding how the awareness can be created 19 respondents mentioned increasing rural exposure of students, while three respondents stated sessions (either lectures or small group sessions) by rural doctors should be conducted. Among other suggestions were sessions on this topic during the orientation program, medical humanities sessions and talks by rural community leaders.

Regarding potential problems which students anticipate while dealing with the rural population fourteen respondents mentioned difficulty in being accepted by the rural community, while eight respondents mentioned that the rural population may be less educated which may lead to problems in their acceptance of treatments and advice provided by the doctor. Eight respondents opined that they did not have much awareness of and exposure to rural life which may lead to problems in adjusting to rural practice. Culture gap, fewer available resources and differences in lifestyle were also mentioned. Racism and language barriers were also mentioned.

Twenty-four respondents mentioned they were aware of initiatives to address the shortage of rural doctors in the US and Canada but specific initiatives were not mentioned. Fifty-seven respondents mentioned they were not aware of these initiatives. Among the initiatives mentioned were debt forgiveness, return of service obligations, flying doctors and encouraging IMGs to practice in rural areas. Fifty-six respondents felt poor return on investment was a factor preventing doctors from working in rural areas while 17 mentioned that this was not a factor. Respondents mentioned recovery from medical school debt requires a job/position which pays well, the world is money oriented, doctors have to study for a long time before they can make money, there are fewer opportunities for making money in rural areas and urban areas provide greater opportunities. A respondent (P 12) stated, “ *Doctors are always in debt after med school. They do not want a poor return. They want progress financially*”. Another respondent (P 21) stated, “ *Payment and financial stability should always be considered when making movements to and from geographical locations*”.

A respondent (P 63) mentioned, “ *This is an interesting study. It is a very important topic, but I fear many people are not as up to date with latest global developments in this area – including myself*”.

Dataset 1, 2 and 3. Demographic characteristics and responses of student respondents Dataset 1: Demographic characteristics of student respondents.
The numbers in the SPSS file have been coded as per the information provided below.
Semester of study: Click here for additional data file. Premed semester 1, 2- Premed semester 2, 3- Premed semester 3, 4- Premed semester 4, 5- MD (undergraduate medical program) semester 1, 6- MD (undergraduate medical program) semester 2, 7- MD (undergraduate medical program) semester 3, 8- MD (undergraduate medical program) semester 4, 9- MD (undergraduate medical program) semester 5 Gender: Male, 2- female Place of family residence; Urban, 2- rural Nationality American, 2- Canadian, 3- others Occupation of father: Health related, 2- others Occupation of mother: Health related, 2- others Dataset 2: Results of the analysis of the demographic data.
Dataset 3: Responses of students to the questionnaire regarding working in rural US/Canada after graduation.

## Discussion

Only about 30% of respondents were from rural areas. Around 41% of respondents were in favor of working in rural US/Canada after graduation. Many respondents however preferred to work in a semi-rural area. Good living conditions, family related factors, welcoming community were among the conditions necessary to be fulfilled. Issues of safety, isolation, lesser potential for personal growth were among the reasons mentioned why doctors were reluctant to serve in rural areas. Difficulty in being accepted by the community, lack of exposure to rural life, cultural issues were mentioned as potential problems for rural practice.

Like in the Nepalese study ^8^, the majority of the respondents were from urban areas. Students from countries other than the US or Canada planned to work in US/Canada after graduation. Caribbean offshore medical schools offer a similar curriculum to US medical schools and students complete their clinical rotations in the US. Most offshore medical schools (OMSs) admit three groups of students a year and provide an increasing contribution to the US health workforce ^9^. Like in Nepal, most respondents willing to work in a rural area preferred a semi urban/semirural area as their work location. The conditions that need to be fulfilled were also similar to the Nepalese study. In Tanzania, increased salaries, hardship allowance, decent housing, good infrastructure, and offering continuing education after a period of service were mentioned as factors making rural jobs more attractive to health workers ^10^. In a survey conducted among healthcare professionals in the Scottish highlands it was found that a rural background, perceived ease of access to children’s education, access to job for spouse and healthcare were important factors influencing rural location ^11^. Professional isolation was an important issue for those working in rural areas.

In a study conducted among occupational therapy students in Australia it was seen that rural fieldwork experience and the influence of fieldwork supervisors were important positive factors for rural practice while the desire to be a specialist, and lack of professional development opportunities in a rural area were negative factors ^12^. The desire for specialization was also mentioned as a negative factor in the present study. In the state of North Carolina in the US incentives such as loan repayment programs, salary guarantees and practice assistance for rural physicians were important factors in attracting primary care physicians to rural areas ^13^. Having grown up in an area with a population of less than 11000 was highly predictive of rural practice. A study conducted in the US found that debt forgiveness, financial incentives and wage guarantees may increase participants’ interest in rural practice ^14^. This was similar to that reported in our study. In the US loan repayment and direct financial incentive programs were successful in recruiting and retaining doctors in needy local communities ^15^.

Family-related factors were mentioned by respondents as influencing their decision regarding working in rural areas. In the US study ^14^ it was noted that medical students with a rural background and with spouses and significant others having a rural background were more likely to practice in a rural area. In a Turkish study it was noted that respondents who were born in an underdeveloped region of the country or had lived for a significant amount of time in an underdeveloped region and with a lower background family income were more likely to work in a rural area ^16^. Higher salaries was the most influential factor for working in these areas. In XUSOM, a large percentage of students, although they are US or Canadian citizens, are of Asian origin. A large percentage of them are from cities and have minimal exposure to rural life and they anticipate problems in being accepted by rural communities.

Medical schools in developed nations with a mandate to create rural doctors are providing a greater proportion of decentralized teaching-learning to students. Students stay in small groups in rural communities and lessons are delivered using the internet and other media. At the University of Calgary in Canada, two separate preclinical programs were taught at two separate rural sites and no significant difference in examination scores were noted between students at the rural sites and students learning at the main university ^17^. At the University of Missouri School of Medicine in the US the summer community program was developed in which second year medical students work alongside rural, community-based physician preceptors ^18^. The program positively influenced student perspective of rural practice and lifestyle and increased their interest in rural practice.

In the US state of Kansas, the Scholars in Rural Health program is designed to attract and retain young rural Kansans with a high probability of successful careers in rural communities. Scholars accepted into and satisfactorily completing this program are admitted automatically to the School of Medicine. The program has shown success in maintaining a pipeline of doctors for rural Kansas ^19^. At the University of Alabama in the US, fifteen years of operation of the rural health leaders pipeline was studied ^21^. Rural students are targeted at multiple levels ranging from elementary school through residency. Puppet shows highlighting different health professions are conducted in elementary schools, a rural health schools program is offered to 11 ^th^ grade students, a minority rural health pipeline program is offered to college students and a rural medical scholars program and assured admissions to family medicine residency are offered to medical students.

A recent article analyzing rural medical education in the US, Canada and Australia identified three types of medical schools, mixed urban/rural schools, defacto rural schools and stand alone rural schools ^21^. These schools all adopted a pipeline approach to meeting the need for rural doctors focusing on: early recruitment; admissions; locating clinical education in rural settings; rural health focus to curriculum; and support for rural practice. In Australia, the Broken Hill University Department of Rural Health was established to improve health care in far-western New South Wales and offers an Enhanced Rural Inter-professional Cultural Health (ENRICH) program to health science students designed to deepen the medical student experience ^22^. The program uses community resources, professional, cultural and artistic to provide stimulating educational resources.

Offshore Caribbean medical schools are privately run for-profit institutions. However, the cost of education at many Caribbean medical schools may be lower compared to their US counterparts. Most Caribbean medical schools at present to the best of our knowledge do not have specific incentives and programs to create physicians for rural areas. A large percentage of our students were from urban areas which may also be true for other Caribbean schools. The US and Canadian governments can offer student loans to their citizens who study in Caribbean medical schools in return for rural practice obligations. At present certain accredited schools are eligible for student loan support. They can also offer residency and other rights to foreign citizens studying in these schools in return for their willingness to practice in rural US/Canada. The Caribbean medical schools can develop closer links with the country’s health system to offer their students increased opportunity for early clinical exposure. Caribbean offshore schools may not have significant financial incentives to produce rural doctors unless specific financial incentives are provided by the US and Canadian governments. We have come across very few studies regarding the rural practice intentions of Caribbean medical students. Further studies in this area are required.

We have not been able to obtain studies regarding the career intentions of graduates of offshore Caribbean medical graduates. A study conducted at the University of West Indies in Jamaica had shown that two-thirds of those who planned to work in a rural health facility planned to do so immediately after graduation but most did not plan to work in these facilities for more than five years ^23^. A study conducted in California had shown that IMG holders of temporary visas had the highest obligation to serve in health professional shortage areas ^24^. A similar phenomenon was noted in the present study with many students from countries other than the US and Canada expressing willingness to serve in areas of physician shortage in return for residency rights. XUSOM also offers scholarships to students from Caribbean community (CARICOM) countries ( http://blog.xusom.com/?p=268). Providing rural exposure on the island of Aruba with well developed infrastructure and health system is challenging. Among the affiliated US hospitals where students do their clinical training most are in urban areas but community hospitals are also included in the list. Students graduating from XUSOM may be interested in serving in rural US/Canada if appropriate incentives and opportunities for professional growth are offered.

The high response rate was the strength of the study. Also a variety of responses were obtained in response to the questions. The study also had limitations. Student perception about working in rural US/Canada was obtained using a questionnaire which had been used in a previous study in Nepal. The questionnaire was modified to suit the present context, and feedback about the modified questionnaire was obtained. The findings of the present study were not triangulated with information obtained using other modalities. Respondents’ awareness about possible changes in medical education and curricula to produce doctors for rural areas and about specific initiatives underway in the US and Canada to address the shortage of rural physicians was low. Though the overall response was high the response to certain questions was low. The study was limited to premedical and preclinical students and was not conducted among students during the clinical years of the course due to logistical difficulties. Seventy-one students from the basic science years participated in the study and the school had around 65 students in the clinical years of study. Their responses were not studied which could affect the generalizability of the study findings.

## Conclusions

Most respondents were from urban areas. About 40% of respondents were in favor of working in rural US/Canada after graduation. Most were however considering working in a small town or a semi-rural area not far from a big city. Like in previous studies good living conditions, family related factors, incentives were among conditions necessary to be fulfilled before students would consider working in rural areas. Most respondents considered reduced opportunities for growth, their lack of exposure to rural life, and cultural issues as hindering factors. Students from offshore Caribbean schools could play a role in partly addressing the physician shortage in rural US/Canada provided the right incentives and growth opportunities are offered. Similar studies are required in other offshore Caribbean medical schools.

## Data availability

F1000Research: Dataset 1, 2 and 3. Demographic characteristics and responses of student respondents, 10.5256/f1000research.5927.d40411 ^25^

## Supplementary file 1

**Student perception about working in rural US/Canada after graduation questionnaire**.

Modified questionnaire used in the study **“Student perception about working in rural US/Canada after graduation”**. Click here to access the File.
