# Supplementary material for: Student perception about working in rural United States/Canada after graduation: a study in an offshore Caribbean medical school
Source: F1000Res. 2015 Apr 23;3:301. Originally published 2014 Dec 10. [Version 2] doi: 10.12688/f1000research.5927.2 (PMC4392823; doi:10.12688/f1000research.5927.2)
Supplement: Dataset 1, 2 and 3. Demographic characteristics and responses of student respondents — Dataset 1: Demographic characteristics of student respondents. The numbers in the SPSS file have been coded as per the information provided below. Semester of study: [file f1000research-3-6852-s0000.tgz › 6db35456-86b9-46ae-a50b-65144b7e0b3c_demographic_characteristics_and_responses_of_student_respondents.zip!/Dataset 3.docx]

**Student responses to various statements in the questionnaire**

**Will you consider working in rural US/Canada after graduation?**

Yes 40 respondents

No 48 respondents

Not sure 10 respondents

**For how many years do you plan to work in rural US/Canada?**

Indefinitely 8 respondents

5 to 10 years 9 respondents

For most of my career 4 respondents

1 to 5 years 4 respondents

Not sure 2 respondents

**What will be your preferred place of work?**

Location does not matter 2 respondents

Semirural 18 respondents

Rural 8 respondents

**What are conditions to be fulfilled before you would consider working in a rural area?**

Good living conditions 20 respondents

Safe rural environment 17 respondents

Proportionately higher wages 17 respondents

Family related factors 15 respondents

The location should be far from a big city 8 respondents

Good working conditions 7 respondents

Acceptance by the community 5 respondents

Prior knowledge of the area 5 respondents

Help with student loans 5 respondents

Provides exposure to different types of patients 4 patients

No racism 2 respondents

Help with relocation 2 respondents

Job security 2 respondents

**Working immediately after graduation or later in your career?**

Immediately after graduation 44 respondents

Later in the career 23 respondents

Not decided 11 respondents

**Why are doctors reluctant to work in rural US/Canada?**

Not enough compensation for the difficulties and challenges faced 27 respondents

Lesser number and diversity of patients 14 respondents

Low standard of living 14 respondents

Less availability of resources 11 respondents

The respondent hails from an urban area 12 respondents

Family related issues 12 respondents

Lack of social ties and activities 11 respondents

Not used to rural life 10 respondents

Greater growth prospects in an urban area 8 respondents

Slow pace of rural life 5 respondents

Isolation 4 respondents

Increased work load 4 respondents

Decreased amount of technology 3 respondents

**How can the government encourage doctors to practice in rural US/Canada?**

Provide greater benefits and salary 57 respondents

Help with repayment of student loans 12 respondents

Upgrade rural hospitals 10 respondents

Create greater opportunities for growth 8 respondents

Educate doctors for practice in rural areas 6 respondents

Make a certain period of rural service mandatory for doctors 4 respondents

During medical school inform doctors about what to expect in rural service 4 respondents

Support doctors during transition and relocation 3 respondents

**Does your medical training adequately prepare you for rural practice?**

Yes 71 respondents

No 19 respondents

Unsure 3 respondents

**Suggestions to refocus medical education for practice in rural areas:**

Teaching students to treat conditions in rural environment 4 respondents

Provide more clinical exposure in rural areas 2 respondents

Train students on how to live in rural areas 2 respondents

Introduce students to complementary and alternative medicine 2 respondents

Have teachers with experience of practice in rural areas 1 respondent

**Are students in the institution aware of problems of life in rural areas?**

Yes 43 respondents

No 39 respondents

**How can this awareness be created?**

Rural exposure 14 respondents

Rural visits 5 respondents

Sessions during the orientation program 3 respondents

Sessions by rural doctors 3 respondents

Talks by community leaders 2 respondents

Medical humanities module 2 respondents

**Problems anticipated in dealing with the local population**

Problems in being accepted by the rural population 14 respondents

Lack of exposure to rural life and rural population 8 respondents

Less education level of the population 8 respondents

Cultural gap 7 respondents

Lack of resources 7 respondents

Racism 4 respondents

Differences in lifestyle 4 respondents

Language barriers 4 respondents

Resistance of the population to change 4 respondents

Lack of health insurance among the population 3 respondents

Large number of patients 3 respondents

Differences in diseases and its presentation 3 respondents

**Awareness about initiatives to address shortage of rural doctors in the US and Canada?**

*Yes 21 respondents*

Use of international medical graduates 2 respondents

Return of service agreements 1 respondent

Debt forgiveness 1 respondent

No 65 respondents

**Awareness about similar initiatives in other countries**

*Yes 24 respondents*

Mandatory rural service 2 respondents

Focus on primary care in the United Kingdom 1 respondent

Flying doctors 1 respondent

No 57 respondents

**Do you think poor return on investment is a major factor preventing doctors from working in rural areas?**

*Yes 56 respondents*

Recovery from medical school debt is important 9 respondents

Less monetary incentive for rural practice 5 respondents

The world is money oriented 4 respondents

Doctors study for a long time before they can make money 3 respondents

Fewer opportunities for advancing career in rural areas 2 respondents

*No 17 respondents*

By practicing in rural areas you will be helping society 2 respondents

Salary is higher in rural areas 2 respondents

Not sure 2 respondents

Not sure 2 respondents
