# Supplementary material for: Student perception about working in rural United States/Canada after graduation: a study in an offshore Caribbean medical school
Source: F1000Res. 2015 Apr 23;3:301. Originally published 2014 Dec 10. [Version 2] doi: 10.12688/f1000research.5927.2 (PMC4392823; doi:10.12688/f1000research.5927.2)
Supplement: Peer review history

# Peer review: Referee response for version 1

**DOI:** 10.5256/f1000research.6335.r7893

**Published:** 2015-04-07

**Authors:** Stacey Friedman

**Affiliations:** 1 Foundation for Advancements of International Medical Education and Research, Philadelphia, PA, USA

This paper addresses the important topic of student perceptions of rural practice, via a questionnaire for students of one Caribbean medical school.

The authors might be more explicit and specific in defining how they are using the term “IMGs from Caribbean medical schools” in this paper since the study participants include students of US, Canadian, and other nationalities, as well as both “premedical” and MD students.

The study’s respondents are diverse, as shown in Table 1 – including “premedical” and MD students, different nationalities, etc.  It would be informative to see more comparison in the analysis, to see if demographic characteristics are related to perspectives on rural practice.  There is some mention of this in the Discussion but it is not detailed in the Results.

It would be helpful if the authors would clarify whether all enrolled premedical and MD students were invited to participate in this study.  The description in the Methods suggests that this is the case but it is not explicitly stated how many students were enrolled at the school at the time of the study, including a breakdown by premedical and MD.  This information would be helpful, as well as confirmation that the study respondents are representative of the overall student population.  Also, some more background on the school, including how it is comparable to and how it differs from other Caribbean schools would be helpful for judging the generalizability of the study results.  Some of this information is in the Discussion.

The authors note that “Many respondents did not answer the question regarding suggestions for re-focusing medical education for preparing doctors for rural practice.”  It would be helpful to know exactly how many respondents there were to this question, particularly given its importance.  It is understandable that the authors would want to be cautious about stating results from this question given the limited response.  However, while stating this caution, the authors may want to offer in the Discussion possible actions that Caribbean medical schools might take to begin to address rural practice needs, including direction for future research.  The low question response rate (despite the high questionnaire response rate) might also be noted as a study limitation.

I have read this submission. I believe that I have an appropriate level of expertise to confirm that it is of an acceptable scientific standard.

# Response

**Published:** 2015-04-13

**Authors:** P Ravi Shankar

**Affiliations:** Xavier University School of Medicine, Aruba

We are submitting the revised version of the manuscript titled ‘ **Student perception about working in rural United States/Canada after graduation’. **The manuscript has been revised in the light of the reviewers’ comments. Response to specific comments is as follows:

S Friedman:  We thank the reviewer for her comments.

   

We have explained what we meant by ‘IMGs from Caribbean medical schools’ on page 3, paragraph 4. The studies we have mentioned from the United States (US) and Canada had defined a Caribbean IMG as either a citizen of the US or Canada who had studied in an offshore Caribbean medical school or students from other countries who had studied at these schools and eventually plan to practice in the US/Canada.             

**Comment**: The study’s respondents are diverse, as shown in Table 1 – including “premedical” and MD students, different nationalities, etc.  It would be informative to see more comparison in the analysis, to see if demographic characteristics are related to perspectives on rural practice.  There is some mention of this in the Discussion but it is not detailed in the Results.

No important difference with regard to perspective about rural practice was noted among different subgroups of respondents. The number of students was small which may have been partly responsible for not detecting differences. Also the study was not designed to statistically examine for differences among subgroups. This has been mentioned on page 5, paragraph 1 (Results section).  

**Comment**: It would be helpful if the authors would clarify whether all enrolled premedical and MD students were invited to participate in this study.  The description in the Methods suggests that this is the case but it is not explicitly stated how many students were enrolled at the school at the time of the study, including a breakdown by premedical and MD.  This information would be helpful, as well as confirmation that the study respondents are representative of the overall student population.  

In the Methods section (page 4) we have clarified that only students enrolled during the basic science years of the MD program and in the Premedical program were included in the study. In the first paragraph of the Results section we have provided the number and percentage breakdown. Students during the clinical years were not included as they do their clinical postings in geographically diverse locations across the US and Canada. This has been mentioned as a limitation of the study on page 10, last paragraph.   

**Comment**: Also, some more background on the school, including how it is comparable to and how it differs from other Caribbean schools would be helpful for judging the generalizability of the study results.  Some of this information is in the Discussion.

We have provided this information on page 3, paragraph 3. As the reviewer has mentioned some of this information is also in the discussion. XUSOM has lesser number of students and a greater proportion of students of South Asian and Asian origin compared to other bigger and older schools. 

**Comment**: The authors note that “Many respondents did not answer the question regarding suggestions for re-focusing medical education for preparing doctors for rural practice.”  It would be helpful to know exactly how many respondents there were to this question, particularly given its importance. 

Only 14 respondents answered this question. We have mentioned this in page 7, paragraph 1.

**Comment**: It is understandable that the authors would want to be cautious about stating results from this question given the limited response.  However, while stating this caution, the authors may want to offer in the Discussion possible actions that Caribbean medical schools might take to begin to address rural practice needs, including direction for future research. 

We have briefly mentioned some of these initiatives on page 10, paragraph 2 of the manuscript.

**Comment**: The low question response rate (despite the high questionnaire response rate) might also be noted as a study limitation.

We have mentioned this in the limitations of the manuscript on page 10, last paragraph.

# Peer review: Referee response for version 1

**DOI:** 10.5256/f1000research.6335.r7546

**Published:** 2015-02-04

**Authors:** Anthony David

**Affiliations:** 1 Department of Physiology, Deccan College of Medical Sciences, Hyderabad, India

The title could be changed a little. The characteristics of the students, rural or urban also was studied and that is not reflected in the title. The study as such is well researched and clear. The findings have been well analyzed and presented in the paper. The abstract reflects the paper well. The explanation of the study design & methods is clear. A little more explanation on the analysis would be useful.

The conclusions are well justified by the results. The data is useful for replication.

Overall this paper can be indexed with a few minor modifications as suggested.

I have read this submission. I believe that I have an appropriate level of expertise to confirm that it is of an acceptable scientific standard.

# Response

**Published:** 2015-04-13

**Authors:** P Ravi Shankar

**Affiliations:** Xavier University School of Medicine, Aruba

Dr David

We thank the reviewer for the comments.

Even though we have studied the demographic characteristics of the student respondents the study was not designed with the objective of comparing perception about working in rural areas among different groups of students. Hence we feel a change of title may not be required as with a change in title readers may expect us to study differences in perception among subgroups of respondents.

We have explained the analysis towards the end of the 2 ^nd^ paragraph of page 4. We used mainly descriptive statistics and numbers and percentages.

We thank the reviewer for the other comments
